# Benefits and Limitations of Text Messages to Stimulate Higher Learning Among Community Providers: Participants' Views of an mHealth Intervention to Support Continuing Medical Education in Vietnam

**DOI:** 10.9745/GHSP-D-16-00348

**Published:** 2017-06-27

**Authors:** Lora L Sabin, Anna Larson Williams, Bao Ngoc Le, Augusta R Herman, Ha Viet Nguyen, Rebecca R Albanese, Wenjun Xiong, Hezekiah OA Shobiye, Nafisa Halim, Lien Thi Ngoc Tran, Marion McNabb, Hai Hoang, Ariel Falconer, Tam Thi Thanh Nguyen, Christopher J Gill

**Affiliations:** a Center for Global Health and Development, Boston University School of Public Health, Boston, MA, USA.; b Department of Global Health, Boston University School of Public Health, Boston, MA, USA.; c Pathfinder International in Vietnam, Hanoi, Vietnam.; d Center for Population Research Information and Databases (CPRID), Ministry of Health, Hanoi, Vietnam.; e Thái Nguyên Provincial Department of Public Health, Thái Nguyên Province, Vietnam.; f Pathfinder International, Watertown, MA, USA.

## Abstract

**Background:**

A randomized controlled trial was conducted in 2015 to evaluate a mobile continuing medical education (mCME) intervention that provided daily text messages to community-based physicians' assistants (CBPAs) in Thai Nguyen Province, Vietnam. Although the intervention failed to improve medical knowledge over a 6-month period, a companion qualitative study provided insights on the views and experiences of intervention participants.

**Methods:**

We conducted focus group discussions (FGDs) and in-depth interviews (IDIs) among participants randomized to receive text messages containing either simple medical facts or quiz questions. Trained interviewers collected data immediately following the conclusion of the trial in December 2015. Using semi-structured question guides, respondents were queried on their views of the intervention, positive and negative, and perceived impacts of the intervention. During analysis, after learning that the intervention had failed to increase knowledge among participants, we also examined reasons for lack of improvement in medical knowledge. All analyses were performed in NVivo using a thematic approach.

**Results:**

A total of 70 CBPAs engaged in one of 8 FGDs or an IDI. One-half were men; average age among all respondents was 40 years. Most (81%) practiced in rural settings and most (51%) focused on general medicine. The mean length of work experience was 3 years. All respondents made positive comments about the intervention; convenience, relevance, and quick feedback (quiz format) were praised. Downsides encompassed lack of depth of information, weak interaction, technology challenges, and challenging/irrelevant messages. Respondents described perceived impacts encompassing increased motivation, knowledge, collegial discussions, Internet use to search for more information, and clinical skills. Overall, they expressed a desire for the intervention to continue and recommended expansion to other medical professionals. Overreliance on the text messages, lack of effective self-study, and technical/language-based barriers may be potential explanations for intervention failure.

**Conclusion:**

As a form of mCME, daily text messages were well-received by community-level health care providers in Vietnam. This mCME approach appears very promising in low-resource environments or where traditional forms of CME are impractical. Future models might consider enhancements to foster linkages to relevant medical materials, improve interaction with medical experts, and tailor medical content to the daily activities of medical staff.

## Abstract (precis)

The original intention was to deliver technical content through brief text messages to stimulate participants to undertake deeper learning. While participants appreciated the convenience and relevance of the text messages, their scores of higher-order knowledge did not improve. The intervention may not have been successful because the messages lacked depth and interactivity, and participants were not explicitly encouraged to seek deeper learning.

## INTRODUCTION

Vietnam, like many countries, is establishing a national continuing medical education (CME) program for health care practitioners.^1^^,^^2^ This effort is embodied in Vietnam's new “Law on Medical Examination and Treatment,” which requires licensure of all clinicians, along with documented CME activities to retain licensure.^3^^,^^4^ It has been supported by large investments such as the Asian Development Bank's “Health Human Resources Sector Development Program” (2011–2015)^5^ and a recent US$106 million World Bank loan to Vietnam's Ministry of Health to help create and manage new CME courses, databases, and monitoring systems.^6^ The success of these activities is critical to help ensure that doctors, nurses, community health workers, and other providers are equipped to provide quality services.

The available evidence indicates that CME can improve knowledge and skills of providers, although data are lacking on the validity, reliability, efficacy, and cost-effectiveness of different CME delivery methods.^7^ While use of new technologies, including electronic, digital, and mobile approaches, appear promising, little research has been conducted comparing traditional CME with these newer delivery strategies.^8^^–^^10^ Three studies that assessed use of short message service (SMS)-based messaging, or text messaging, for CME purposes suggest that such approaches are feasible and may be useful for distance learning, but the studies provide little insight into how to use text messages to maximum advantage.^11^^–^^13^ While not focused on CME, a recent systematic review of eLearning approaches for undergraduate medical education in low-resource settings published by the World Health Organization highlights potential advantages—cost-savings, scalability of educational materials, freed-up instructor time, ease of developing and updating content, and portability—that might extend to CME provision.^14^ It also summarized benefits described by learners that may offer insights into features affecting learning: ease of access, flexibility, improved student-teacher contact and discussions, and more exchange with peers.^14^

CME can improve knowledge and skills of providers, but data are lacking on the effectiveness of different delivery methods.

To provide evidence on a potentially scalable and cost-effective CME approach in Vietnam, our Boston University (BU)-Pathfinder, Inc. team collaborated with Vietnam's Ministry of Health to assess a text message-based CME intervention, the results of which have been previously reported.^15^ Because most clinicians in Vietnam practice at the community level, we focused on providers working at community health stations (CHSs), known as community-based physicians' assistants (CBPAs). The trial was implemented in Thái Nguyên, a rural province north of Hanoi, with the Thái Nguyên Provincial Department of Public Health. A total of 638 CBPAs were randomized to 1 of 3 arms: group 1 (control arm) received a weekly non-medical SMS message; group 2 (passive intervention arm) received a daily medical SMS message related to primary care, with a reply requested using any combination of letters and numbers; and group 3 (interactive intervention arm) received a 4-option multiple-choice SMS question (covering the same themes as the messages sent to group 2), with an answer requested and an immediate reply provided indicating whether the answer was correct. Message content was based on the content of CBPAs' original training curriculum covering 6 topic areas: surgery, internal medicine, pediatrics, infectious diseases, sexually transmitted infections, and family planning. Messages were delivered in random order rather than being organized by topic.

Our hypothesis was that the intervention, by delivering daily messages over a 6-month period on previously learned topics, would provide 2 pathways to improved knowledge: (1) learning from the SMS messages themselves (weak pathway), and (2) motivation to increase “lateral learning,” or self-study (strong pathway). Given evidence that interactive approaches are better liked and more effective in improving knowledge,^16^^–^^19^ we also anticipated that group 3 (whose members answered quiz questions and received subsequent responses) would be more motivated than group 2. We assessed impact by comparing post-intervention exam scores (percentage of questions answered correctly) between groups and within-group pre-and post-intervention scores, using typical multiple-choice questions designed around the 6 topic areas, in line with CBPAs' original clinical training. The exam questions were similar to the daily quiz questions provided to group 3, although they were more complex than the quiz questions. That is, the quiz questions required a simple answer to a direct question, such as ‘*What is the optimal antibiotic for streptococcal pharyngitis*?' (answer: amoxicillin). The exam questions required knowing 2 or more medical facts to answer. For example, a typical exam question was worded as such: “*A child with a sore throat developed a macular rash after taking an antibiotic. What was the most likely cause of the sore throat?”* (answer: mononucleosis.) To answer this exam question correctly, the participant required knowing the differential diagnosis of sore throats, the most likely treatment (amoxicillin) for streptococcal pharyngitis, and the fact that macular rash is a common result of using amoxicillin in the setting of mononucleosis. The exam questions were not designed to be answerable from knowing the correct answers to the quiz questions alone; rather, they assumed further knowledge above and beyond the content of the quiz questions. A detailed description of the trial's methods and results is available in the main effects paper, published in November 2016.^15^

One component of the project, the focus of the present study, was a qualitative exploration focused on the experiences of trial participants. Data were collected immediately following the trial while the intervention experience was fresh in the minds of CBPA participants. The main study questions were: (1) What were trial participants' views of the intervention, both positive and negative?, and (2) What were the perceived impacts of the intervention?

After completing data collection, we learned from the trial results that the intervention did not achieve the primary goal of increasing medical knowledge. As reported in the main effects paper, we found that mean test scores varied from 36.1% to 39.0% at baseline and from 40.1% to 40.9% at post-intervention, with no significant differences between arms at either point or within arms over time, meaning that the intervention did not succeed in its primary objective of improving medical knowledge.^15^ However, the results also showed that the intervention was well-received by CBPAs in terms of participation rates, technical feasibility, and potential cost-savings, provided it could be effective. Given support for a second mobile continuing medical education (mCME) trial, we wanted to understand how we might modify our approach to improve the likelihood of a positive effect. We thus added a third study question in the analysis stage: Were there features of participants' experiences that could help us understand why the intervention failed?

We conducted a qualitative study to learn how to modify a text message-based CME program to improve the likelihood of having a positive effect on providers' knowledge.

## METHODS

### Study Site and Participants

Study respondents were CBPAs who participated in the mCME trial, and thus had met enrollment criteria at baseline. They were 18 years or older and provided primary care services in Thái Nguyên. Each had completed high school, had graduated from an accredited 2-year medical training program, and owned an SMS-enabled cell phone. Given our focus on intervention experiences, respondents were limited to group 2 (passive intervention) or group 3 (interactive intervention) trial participants. Respondents engaged in a focus group discussion (FGD) or an in-depth interview (IDI).

### Design and Sampling

In light of time and budget constraints, we elected to conduct a total of 8 FGDs, with 1 FGD for trial participants within each of 8 categories, stratified by study arm (group 2 vs. 3), gender (male vs. female), and SMS response rate during the trial (high, which we defined as >90% among women and >75% among men, vs. low, defined as <10% among women and <25% among men; these proportions differed due to the distribution of high vs. low response rate by gender^15^). These groupings were designed to ensure collection of a range of perspectives, particularly because response rate could be interpreted as a proxy for intervention enthusiasm. A total of 8 participants in each group were selected randomly and invited to participate in an FGD by written invitation from collaborators from the Thái Nguyên Provincial Department of Public Health. In each group, there were an additional 6–10 participants whom we identified as backups for FGDs and for IDIs. One participant within each group was identified randomly for IDI participation. We also purposefully identified 7 participants who had displayed various challenges responding to texts during the trial for offer of participation, thereby providing a total maximum sample of 15 participants for IDIs.

### Data Collection

Data were collected in December 2015. Before data collection commenced, members of the study team held a training workshop in Hanoi for 8 local interviewers. The workshop focused on the protocol, qualitative research methods, and protection of human subjects in research. We reviewed the question guides to ensure their clarity and appropriateness of questions and probes and engaged in role play to sensitize local interviewers to issues that might arise during data collection.

During the FGDs and IDIs, interviewers worked in teams of 2, with one asking questions and the second one taking notes (to clarify potentially confusing responses, document non-verbal reactions, etc.). Discussions were conducted in Vietnamese, at the Thái Nguyên Provincial Department of Public Health in Thái Nguyên City. Interviewers used semi-structured question guides that queried specific topics yet allowed for open-ended responses and follow-up probing. Each FGD and IDI was audio-recorded and took 60–90 minutes to complete. Respondents were not compensated but received snacks.

Questions focused on views of and experiences related to receiving CME via text messages, and, for respondents assigned to group 3, the daily quiz questions. We queried whether and how the intervention might have impacted attitudes and behaviors; we also asked about the SMS intervention compared with traditional forms of CME, and what respondents thought were the benefits and drawbacks to using text messages for CME.

### Data Analysis

The FGD and IDI recordings were transcribed verbatim and then translated into English. Boston-based team members analyzed transcripts in QSR NVivo 11. Transcripts were read, with themes and subthemes identified and cross-checked by multiple readers. We created a theme codebook, which was used to code each transcript. The analysis included comparing FGD and IDI responses; we also examined responses by group assignment and gender. We prioritized responses by their frequency and also explored divergent views. Responses were included whether mentioned spontaneously or in reply to a follow-up probe.

### Ethical Review

The study was reviewed and approved by the institutional review boards at Boston Medical Center and The Hanoi School of Public Health. All respondents provided written informed consent.

## RESULTS

### Background Characteristics of CBPA Participants

We conducted 8 FGDs and 15 IDIs, with a total of 70 respondents. Between 5 and 8 CBPAs participated in each FGD. One-half of the respondents were men, and the average age of all respondents was 40 years (Table). About half (49.3%) were former group 2 participants. Of FGD respondents, about one-half (52.7%) had high response rates during the trial; among IDI respondents, 7 had “medium” response rates, while 4 each were in high and low response strata. The majority (81%) of all respondents practiced in rural settings; only 3 were city-based. Most (51.4%) focused on general medicine; fewer worked in obstetrics and pediatrics (20%), traditional medicine (21.4%), or preventive medicine (7.2%). The mean length of service was 3 years.

**TABLE.** Background Characteristics of mCME Participants, Thái Nguyên, Vietnam, December 2015

	IDIs (N=15)	FGDs (N=55)	Total (N=70)
Gender, No. (%)			
Female	8 (53.3)	27 (49.1)	35 (50.0)
Male	7 (46.7)	28 (50.9)	35 (50.0)
Age, mean (SD), years	38.6 (10.5)	40.8 (11.2)	40.3 (11.0)
Study arm, No. (%)			
Group 2: medical facts	4 (26.7)	30 (54.6)	34 (49.3)
Group 3: medical questions	11 (73.3)	25 (45.4)	36 (50.7)
Response rate during study,^a^ No. (%)			
High	4 (26.7)	29 (52.7)	33 (47.1)
Medium	7 (46.6)	0 (0.0)	7 (10.0)
Low	4 (26.7)	26 (47.3)	30 (42.9)
Practice setting, No. (%)			
Rural	12 (80.0)	45 (81.8)	57 (81.4)
Town	2 (13.3)	8 (14.6)	10 (14.3)
City	1 (6.7)	2 (3.6)	3 (4.3)
Medical specialty, No. (%)			
General medicine	8 (53.3)	28 (50.9)	36 (51.4)
Obstetrics and pediatrics	3 (20.0)	11 (20.0)	14 (20.0)
Traditional medicine	3 (20.0)	12 (21.8)	15 (21.4)
Preventative medicine	1 (6.7)	4 (7.3)	5 (7.2)
Years in health sector, mean (SD)	2.7 (0.4)	2.9 (0.5)	2.9 (0.5)

Abbreviations: FGD, focus group discussion; IDI, in-depth interview; mCME, mobile continuing medical education; SD, standard deviation.

a High response rate refers to those in the 90th and 75th percentiles for women and men, respectively; medium refers to those between the 10th and 90th percentile for women and between the 25th and 75th percentile for men; low refers to those in the 10th and 25th percentiles for women and men, respectively.

#### Views of the Intervention

All respondents, regardless of group assignment or gender, communicated positive attitudes and experiences with the intervention. The most common adjective used to portray the mCME text messages was “useful,” with expanded descriptions including “incredibly helpful,” “very good,” “very effective,” and “very useful for us CBPAs.” Detailed reactions coalesced around the text messages' convenience and relevance. Some respondents noted drawbacks to the messages. The vast majority wanted the intervention to continue. These themes are explored in more detail below and summarized in the Figure. We note differences by gender, group, and data source (IDI vs. FGD), with illustrative statements provided.

**FIGURE:**
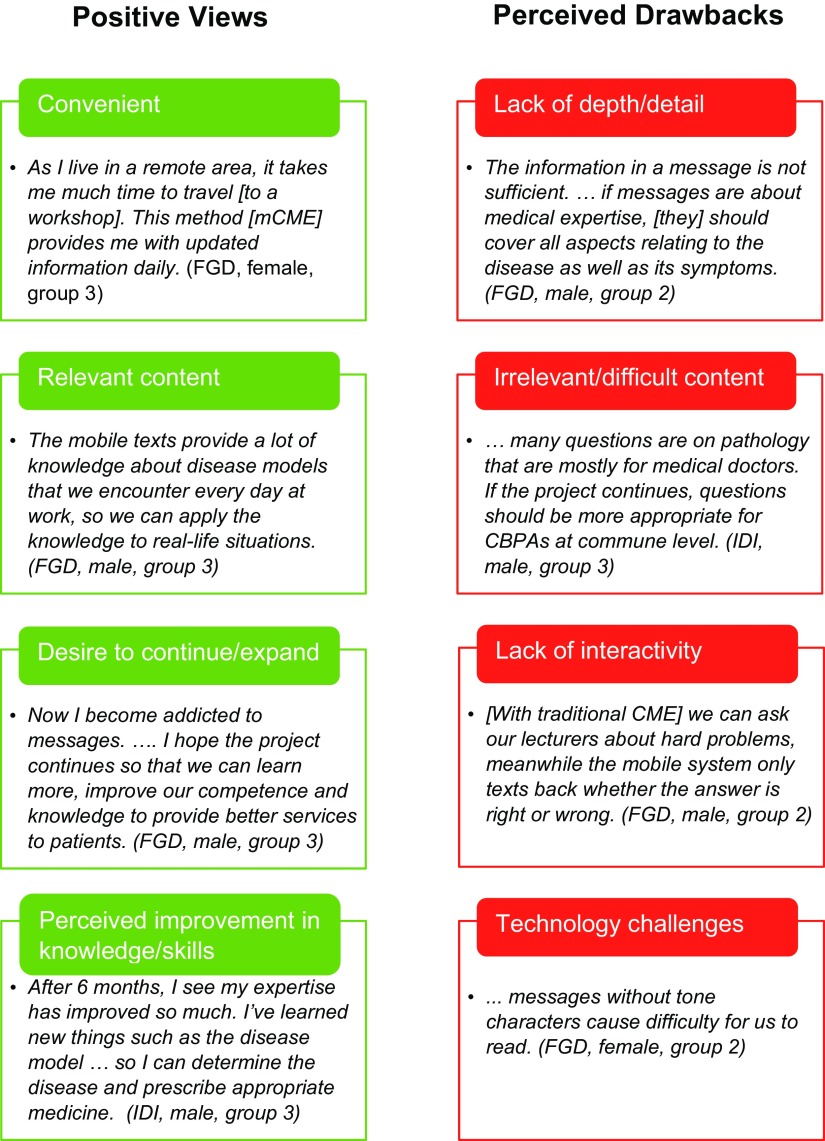
Participant Views of the mCME Intervention With Illustrative Quotes, Thái Nguyên, Vietnam, December 2015 Abbreviations: FGD, focus group discussion; IDI, in-depth interview; mCME, mobile continuing medical education.

##### Convenience

Virtually all respondents highlighted the convenience of the mCME approach, noting that the text messages could be accessed anytime, anywhere, with responses submitted later. They liked receiving messages at work, where content could be discussed with colleagues. Many highlighted the succinct nature of messages (which had length restrictions), their comprehensibility, daily arrival, and ease of phone-based access and storage. One typical statement was:

*This new method makes use of our free time, and doesn't force us to go any place to study … we can study one day after another steadily.* (Male FGD participant, group 3)

Most respondents contrasted the texts' convenience favorably with traditional CME approaches. They highlighted the time and expense of workshop-related travel and attendance, which often were insurmountable barriers. As one male FGD participant (group 2) stated:

Through this method, many people can learn and access information at the same time, while [with] traditional training, it's hard to organize for us because we are experienced CBPAs and [work long hours] so we cannot participate in 3–6 month training courses just to update our information. Who will take over our tasks …?

Many compared the ease of phone-based information with the bulky books and materials common to workshops. Others emphasized that text messages did not interfere with their work, lauding their focused, daily information:

*I can remember if I learn just a bit every day, but it's impossible if I have to stay in a class all day long from morning ‘til night! I'm very satisfied with this approach.* (Female FGD participant, group 2)

One mentioned that traditional CME was stressful due to the intensive studying and testing involved, whereas texts were “… *nearly stress-free*.”

Several respondents were circumspect about the text messages' relative advantages:

*For mCME, firstly it's fast; secondly it saves time; thirdly, it doesn't interfere with work. But the traditional training method has its advantages as well; the written materials have detailed tables of content so I can easily find what I want to review if I forget.* (Female FGD participant, group 3)

A handful noted that, although convenient, phone-based information entailed risks:

*We think that the questions are very useful, but it's easy to lose them. One day I went to a shop to repair my phone, and the technicians deleted all my texts in my inbox. I couldn't find the medical texts anymore, not even one left. All gone.* (Female FGD participant, group 3)

Nearly all respondents highlighted the convenience of the mobile CME approach.

##### Relevance

Most respondents found both forms of messaging, simple medical facts and quiz questions, appropriate for CBPAs. The text messages were described as “very suitable” and “relevant.” One male FGD participant (group 2) summarized:

Many questions provide knowledge relevant to the diseases at my work—it's so useful.

Others stressed the texts' practicality and application “… *to real-life situations*.” Several mentioned that message content received on one day had been helpful when treating a patient on the very same or next day.

Relevance was linked frequently to the wide range of topics covered in the text messages. Respondents claimed that, as community-level primary providers, they required understanding of numerous topics, unlike higher-level providers, who usually specialized in one field. Many noted the value of information on specific conditions—mentioning specifically tuberculosis, HIV, Parkinson's disease, gynecological diseases, low weight and malnutrition in children, diarrhea, obstetric complications, bleeding problems, and children's choking—and in different medical areas, including symptoms, diagnosis, prevention, and treatment. Several contrasted the text messages' breadth with conventional CME:

*I think this approach [mCME] provides wide and diverse medical knowledge that could not be fully covered in traditional training sessions.* (Female FGD participant, group 3)

While respondents liked both text formats (daily medical facts and daily quiz questions), they revealed a clear preference for the latter. Quiz questions were seen as intriguing and motivating, and respondents liked receiving quick “right or wrong” responses. As one FGD female participant (group 3) explained:

Since feedback to our answers is immediate, we know which questions are correct or wrong, so we would know if we make mistakes or not.

While most group 2 FGD participants said that they benefited from medical facts alone, others desired feedback:

*But it'd be better to have interaction with the system like reading questions and then answering and waiting for the system's feedback, rather than only replying.* (Female FGD participant, group 2)

In one FGD, a group of men agreed that they liked the quiz format best:

As Group 2, we receive the statement about the symptoms … but the other group can answer the question. It's more interesting if you can join quizzes.

Respondents liked to receive both medical facts and quiz questions via text message, but had a clear preference for the interactive quiz questions.

##### Drawbacks

The most frequently mentioned disadvantage of the text messages was lack of depth. Although most respondents liked the texts' brevity, one-half (slightly more in FGDs than in IDIs) noted that the texts lacked detail. As one male FGD participant (group 2) stated:

… the SMS's content is insufficient; [it's] so short and limited.

The most frequently mentioned drawback of the text messages was lack of depth.

Several suggested that text messages should provide not just more information but information delivered in a logical order:

*Today you send the symptoms of one disease, diagnosis tomorrow, and treatment after that.* (Female FGD participant, group 2)

Just over one-third of respondents indicated that some texts were either irrelevant or difficult. Most typically, they referred to messages about diseases or procedures that CBPAs do not normally encounter or perform, such as lab tests. A smaller group found some text messages very difficult to understand. This is recounted from a FGD with men, all from group 3:

Participant A: *The education level of this project is for doctors or for whom? Because there are many questions even doctors at my CHS aren't able to answer.*

Participant B: *A lot of questions are beyond my knowledge; CBPAs at communes like me can't answer.*

Participant A: *That's why I want to ask, what is the purpose of producing those hard questions? Who are they for? I know this project is for education, but who is it trying to educate?*

In addition, about one-third of respondents indicated that lack of interaction—particularly the inability to ask questions—was a limitation. They admitted readily that, while convenient, the simplicity of text-based communication could be frustrating. One male FGD participant (group 3) explained:

There's no one to answer my questions if I don't understand.

Many respondents noted the lack of interaction as a limitation to the text messages.

Several offered suggestions for incorporating feedback; as a female FGD participant (group 2) recommended:

It would be better if the system may send us the questions/SMS and then ask if we understand it/how we feel about it, [do you have] any questions about this issue, etc.

Finally, about a third of FGD participants and half of IDI respondents described challenges with technology. This included challenges with cell phone use generally and/or poor reception; more found it difficult to read texts without the phonetic symbols common to written Vietnamese, or to respond to texts. As one male FGD respondent (group 3) stated:

… messages should be written more precisely with tone marks, so that we do not have to think hard.

In response, his FGD companions explained that they shared confusing texts with other CBPAs, who helped them decipher messages, so opinions differed on this point. Another subset of respondents struggled to respond correctly to the text messages. A female IDI respondent (group 3) described her experience:

At the beginning … I wrote ‘Answer A, B ….’ I also sent responses like ‘Answer A is correct …,’ and the system [responded that this '] was incorrect. It's true that I was very disappointed …. Later on, I was provided with an explanation on how to send responses. I became more used to it and found it very interesting.

##### Continuing and Expanding mCME

Virtually all respondents expressed a desire for the intervention to continue. Several said they had felt nervous initially but had become accustomed to daily messages and would miss not receiving them. As one male FGD participant (group 3) claimed:

Once we get used to it, we will like it. Now I no longer receive messages. I feel like I'm losing knowledge.

Some recommended a year-long timeframe; others stated more generally that they would prefer the training to last longer or to be implemented continuously. One male FGD participant (group 3) claimed that he was “addicted” to the texts. Some stated that the texts were so valuable they would pay themselves for continued delivery.

Similarly, most respondents believed that mCME should be expanded to other health care providers. The most common view was that nurses, doctors, and midwives could also benefit from text-based information. Several also reiterated the importance of tailored content:

*I'd like this approach to expand to doctors and nurses as well, with levels of questions suitable for each of the profession.* (Female FGD participant, group 3)

### Perceived Impacts of the Intervention

Respondents described numerous impacts they believed stemmed from the intervention. Many emphasized that just being the focus of an educational intervention had improved their outlook regarding their work. As one male FGD participant (group 3) noted:

We need to receive more knowledge … this type of education is very good. It helps change our attitude.

More substantively, respondents described impacts that encompassed improved knowledge, confidence, and skills; increased Internet use; and more discussion of medical issues at the CHSs. Some felt that the intervention had made them better CBPAs. We explore these themes in more detail below.

#### 

##### Improved Knowledge

Virtually all respondents indicated that the intervention had improved their knowledge—or that it had such potential. Increased knowledge was linked to accessibility, understandability, and frequency of the text messages:

*Each day, we can gain more knowledge. Messages are sent daily so we can remember them more effectively … .* (Male FGD participant, group 2)

Many believed that the texts rekindled a desire to learn. Several stressed their ability to motivate lateral learning:

The best thing we get is that the project promotes us to think and learn new knowledge or improve old knowledge. (Male FGD participant, group 3)

A major theme was the role of the text messages in helping respondents relearn material. As an IDI male respondent (group 2) stated:

The most useful thing is that I'm reminded of past knowledge that I've learned at medical school.

Many referred to their lengthy service as providers, lamenting their lack of CME opportunities. Some admitted to long-forgotten knowledge. This statement was typical of older participants:

*Younger people have high qualifications, but I graduated 30 years ago so my knowledge and skills are faded.* (Male FGD participant, group 2)

A sense that daily messages provided important support and prompted a desire to learn was conveyed by a number of respondents, as in this statement:

*It has been 20 years since I [have been] work[ing] as a CBPA. At the local level, there's not much training and refresher training. Since I joined this type of education, I feel like I have received much help. When I received messages, I had to study hard and then tried to give correct answers. … From this project, I gained more knowledge.* (Male FGD participant, group 3)

Respondents also stressed the new knowledge they believed they had gained. Although some felt that certain messages were irrelevant to their work, the majority expressed appreciation for the chance to learn new things, an important opportunity for some given the passage of time since their training:

*After 20 years, books are also out of date, partly because of new knowledge. This type of training helps us revise what we have learned. It can replace a teacher. In addition, there are things we haven't learned or we just know a little about. This training provides us with more knowledge.* (Male FGD participant, group 3)

Respondents thought they had gained new knowledge from the text messages.

When queried about quiz question feedback (telling them whether their answer was correct), most group 3 FGD participants claimed they were sad or disappointed at receiving “incorrect” feedback, yet learned more from such responses. As one female IDI respondent elaborated:

*I think the wrong answers are very useful, especially when I discover that, ‘Ah, I answered this question wrong. This one is the correct answer.’ Through each wrong answer, I can learn a new lesson ….* (Female IDI respondent, group 3)

Others highlighted the way that such feedback motivated them:

*But when I answer incorrectly, I study more, use my brain more, such as looking up books, asking colleagues or using other methods to find out where I was wrong, and then I note it down or else I would forget.* (Male FGD participant, group 3)

##### Increased Self-Study and Discussion

Additional major themes were perceived changes in lateral learning (self-study) and in discussion with colleagues about medical issues. Many respondents communicated that the text messages prompted them to look things up, to study, and, especially, to use the Internet to search for more information. For some, because the information provided in the text messages was necessarily limited, the text messages provoked a craving for more information, as explained by a male FGD participant (group 2):

The SMS is short, but it encourages us to find more information online. It's helpful to motivate us to learn more.

As noted earlier, some respondents linked such motivation to the feedback received from the mCME program on an incorrect quiz answer. This statement by a female IDI respondent (group 3) illustrates this point:

Before, I rarely went on the Internet, I just read medical books. But since participating in the project, I often go online to search for information when I get the answers wrong.

Many respondents recounted how the text messages had spurred discussions at their CHSs. They described asking their colleagues, including doctors, specific questions about the information included in a text message. Several expressed delight that the texts seemed to stump their senior colleagues. Others described animated debates that took place among colleagues; some claimed camaraderie among the CBPAs was enhanced from the intervention. As one male FGD participant (group 2) noted:

Staff in healthcare station receive messages at 9 or 10; they will discuss [the texts] with each other, so the atmosphere in the CHS is very good.

Many respondents recounted how the text messages had spurred discussions with their colleagues.

##### Enhanced Confidence

The vast majority of respondents claimed that the intervention had improved their self-confidence. As one female FGD participant (group 3) explained:

I feel that I have more knowledge, I'm more confident in my expertise.

This reaction was common among group 2 participants as well:

*During 6-months of receiving SMS, I feel more confident because of [improved] professional knowledge. I have studied those [points] but forgot them …. I can improve my knowledge and feel more confident in handling daily tasks.* (Female FGD participant, group 2)

##### Improved Competence

A number of respondents perceived improved clinical skills. Most frequently, they described an enhanced ability to diagnose conditions. Several spoke generally of improved technical competence. In the words of a female FGD participant (group 3):

With this project, I know more symptoms. There are cases that I am not sure [about], but now I am confident. With the knowledge I receive, I see my technical competence is improved.

Others relayed that their job performance was better than before:

*From this project, I get more knowledge. It's very good and useful. … That greatly improves my job.“* (Male FGD participant, group 3)

### Why Did the Intervention Fail?

Several themes are suggestive of why the 6-month intervention failed to show improved medical knowledge. These encompass overreliance on text messages for medical information, lack of effective self-study, and specific issues with technology and messages. Each is explored below.

#### 

##### Exclusive Focus on Text Messages as “Professional Handbook”

Many respondents seemed to view the text messages as the main, if not sole, source of medical information, rather than as a stimulus to further learning. As discussed earlier, they liked the texts largely because of their simplicity and brevity; this convenience may have encouraged them to focus on the texts exclusively in lieu of exploring more detailed materials, whether book- or Internet-based, which might have helped them to score better on the endline exam. As one male FGD participant (group 2) relayed:

In the traditional training, it's different. We need a certain period of time to sit down and study, while this new method is much more convenient; we only need to open our phone and read text messages.

Many respondents viewed the text messages as the main, if not sole, source of medical information.

In addition, the random sequence of the texts may have hindered learning. Thus, while respondents said that the text messages prompted self-study, the predominant behavior appears to have been text message-focused, with the content of the text messages raised to an exalted status, as conveyed in this statement:

*I consider the texts as a professional handbook.* (Female FGD participant, group 2)

It is also possible that traditional modes of learning in Vietnam, which stress memorization rather than thoughtful inquiry, reinforced a tendency to simply memorize the text messages rather than to use them as a springboard to meaningful learning. Many respondents described reviewing the text messages, repeating their ease of access. Said one respondent:

*In the evening at home I'll read the messages for an information update. It would be much harder to review books or professional materials.* (Female FGD participant, group 3)

Memorization, rather than a probing approach, may also have limited deeper learning from other medical sources.

##### Lack of Meaningful Self-Study

As discussed earlier, many respondents described changes in behavior, particularly increased Internet use and collegial discussions, that could be expected to lead to increased knowledge. Since we know knowledge did not improve measurably, we can suppose that any actual behavioral changes were ineffective pathways to learning. Thus, when a respondent said, ”I often go online to search for information when I get the answers wrong,“ that alone does not mean that accurate information was obtained or that something was learned. Similarly, increased CHS-based discussions prompted by the text messages cannot ensure meaningful education. Indeed, many respondents noted that they took unclear content to their physician colleagues, but often without a helpful result. As one male FGD participant described:

*I've discussed the questions with specialized doctors and MAs [Masters degree holders]; sometimes even they answer incorrectly.* (Male FGD participant, group 3)

##### Technology and Content Challenges

As indicated above, some respondents experienced technology challenges and/or found the content in the text messages challenging to understand. While this does not seem to have stifled generally positive views of mCME, it may have impacted behavior during the trial to a greater degree than respondents themselves realized. Several suggested that if CBPAs encountered difficult text messages, the text messages might be ignored. A female IDI respondent (group 3) elaborated, as follows:

Sometimes, using difficult words made them unable to understand and answer, which would reduce their motivation. … they might think that the questions were not relevant with their daily work, so they might give up.

## DISCUSSION

This qualitative study sheds light on participants' experiences during a 6-month trial that assessed a novel approach to CME in a low-resource setting—using mobile technology to deliver daily text messages to community-level health providers in Vietnam. We found mainly positive views of two forms of mobile messaging (passive and interactive); respondents highlighted convenience, relevance, and feedback, as well as perceived benefits including increased motivation, knowledge, collegial discussions, Internet use to search for more information, and clinical skills. They also described downsides to the text messages: lack of depth, inability to interact, technical difficulties, and challenging content. Overall, they reacted positively, and expressed a clear desire for the mCME intervention to continue and to be expanded to other medical professionals. Given the potential of mHealth approaches to reshape the CME landscape and the lack of evidence on effective strategies to provide mCME,^1^^,^^7^ these findings provide important evidence that can inform future mCME research and implementation efforts.

The hypothesis for the trial was that daily text messages would motivate lateral learning and lead to improved knowledge, as measured by post-intervention exam scores. This did not occur, and the present qualitative study's findings help illuminate why it did not. First, respondents expressed an extraordinary focus on the text messages themselves. Yet the messages were, by design, limited in content and not organized with intensive studying in mind. To the contrary, they were meant to motivate CBPAs to seek out and use more detailed sources of medical information. With the benefit of hindsight, we postulate that our lack of clarity with trial participants about the goal of self-study and the exam content not being solely based on the text messages, along with the possible lack of easily accessible, reliable medical materials, may have played a role in extensive focus on and use of the text messages. Because the exam questions were more complex than the quiz questions, a strategy of focusing on the text messages alone was unlikely to be successful in terms of performing better on the endline exam compared with the baseline exam. It is also possible that daily practicalities drove CBPAs' behaviors. Previous research on eLearning has shown that users value ease of access and flexibility.^14^ Respondents in this study prized convenience; it may be precisely their easy access and flexibility that made the text messages so irresistible, perhaps fostering an illusion of and satisfaction with “studying” by reviewing the text messages, but not deeper learning. Participants were also receiving text messages in a busy environment (their clinics); multitasking may have hindered full absorption and later follow-up.

While respondents' accounts of changes in knowledge, confidence, and study habits are encouraging, our trial results confirmed that such changes, where they occurred, did not result in increased medical knowledge. As discussed above, Internet searches and collegial discussions do not translate automatically into effective learning. Indeed, given that respondents revealed a strong disinclination toward traditional and intensive forms of CME, Internet searches and discussions may have supported a tendency to avoid serious study and engage instead in lighter activities that, again, provided impressions of learning without meaningful gains in knowledge. Additionally, given that some time had passed since formal training for many CBPAs, and that some may have lacked direct, convenient access to reliable material, it is possible that focused and effective self-study was simply very hard to achieve.

To address some of these issues, our next mCME project will be more transparent about behavior-change aims and will incorporate links to existing Internet-based CME courses in the text messages. With a focus on HIV providers in Vietnam, our goal will be to provide participants with accessible, reliable medical sources to supplement the information received in the text messages. In this way, we hope to discourage the temptation to focus only on the text messages and to support the use of existing relevant continuing education. Because the Vietnamese government has made an enormous investment in online courses, this also serves to support the country's long-term CME goals.^1^^,^^2^^,^^5^

It is also critical to consider creative approaches to overcoming the downsides of text-based CME while retaining its strengths. Again our qualitative data are informative. Respondents' affirmative attitudes toward mCME centered on its convenience; their perspectives also underscored the role of relevant content, feedback, and interaction in motivating professionals—qualities previously identified as important in CME.^7^^,^^20^ Respondents themselves suggested that relevance could be enhanced by tailoring content more precisely for professionals in different medical fields. They also recommended that messages be organized in a sequence that mirrors clinical encounters (symptoms, diagnosis, treatment), although clearly there is room for experimentation, in both the substance and sequencing of content. Another approach might be to add a component on searching and finding quality information on the Internet. These endeavors are time-demanding but certainly possible technically.

Addressing the text messages' lack of depth and inability to interact is more complicated, since this should be done without compromising simplicity. Several approaches come to mind; further research would help examine their potential promise. One is to add optional additions for those who desire more, with opt-out choices for those who do not. First, for more depth, links to further online information and courses by topic and to in-person courses, could be incorporated into the text messages (as we are now doing); these might be complemented with other resources (fact sheets, infographics, Internet-based eLearning sessions, etc.). Second, for more interaction, daily text messages could be supplemented by optional weekly group chats, call-in numbers to a hotline staffed by a professional, and web-based, interactive workshops. Third, a champion or expert on-site might be identified to lead discussion groups. Fourth, text- or computer-based quizzes could be added to the end of specific modules (say, after 1 or 2 weeks) so that participants can assess their own learning immediately after finishing a sequence of texts. The ability to ask questions, obtain immediate feedback, and observe progress over time might well substantially enhance the appeal of the basic text-based mCME package assessed in this first trial. While any of these could be stand-alone options, a combination of options could also be incorporated into a fixed package. The goal is the same: keep the simplicity and convenience but create room for depth and interaction.

To improve interactivity, text messages could be supplemented by group chats, hotlines, and web-based workshops.

### Limitations

We acknowledge limitations to this study. First, some respondents may have provided biased information—whether from a desire to please facilitators (moderator-acceptance bias); to avoid expressing conflicting views in the company of respondents who held strong opinions (dominant-respondent bias); or because of poor recall. Since many respondents provided a range of views, we do not believe such potential bias is concerning. Second, we collected data from a limited number of participants in a specific experiment; the views reported here are not generalizable beyond CBPAs practicing in Vietnam. That said, our goal was precisely to learn about the reactions to such participation to improve on this initial mCME experiment. Given the potential scalability and cost-effectiveness of mCME approaches, we believe these findings are important in contributing to understanding about the strengths and weaknesses of text-based learning and suggest possible paths forward for work in this important field.

## CONCLUSION

This qualitative study found predominantly positive reactions from participants in an mCME trial in Vietnam, where efforts are underway to expand CME to health professionals. Participants provided positive feedback on the text message-based intervention and were enthusiastic about its perceived convenience, relevance, and motivating effects. However, the results confirmed that text messages alone cannot stand on their own; they require a framework to translate motivation into meaningful behavior change. They also underscore the higher appeal and superiority of interactive approaches in engaging learners. If adapted appropriately for different settings and medical professionals, mCME could be a promising tool for distance learning.

## Notes

**Peer Reviewed**
